# Draft genome of *Streptomyces* species VRG-4 isolated from leaf cutter ants of Uttarakhand

**DOI:** 10.1128/mra.00890-26

**Published:** 2026-08-13

**Authors:** Vanshika Rana, Bindu Naik, Sanjay Gupta, Vijay Kumar

**Affiliations:** 1 School of Biosciences, Swami Rama Himalayan University 422223, Dehradun, Uttarakhand, India; 2 Department of Food Science and Technology, Graphic Era Deemed to be University 209257, Dehradun, Uttarakhand, India; Michigan State University, East Lansing, Michigan, USA

**Keywords:** *Streptomyces tendae*, leaf cutter ant, actinobacteria, biosynthetic gene clusters

## Abstract

We describe the draft genome sequence of *Streptomyces* sp. VRG-4, isolated from leaf cutter ants (Uttarakhand, India). The genome (9.3 Mb) consists of 83 contigs with 72% GC content. Genome annotation identified 8,243 protein-coding genes and 69 tRNA genes, which can be further used for functional and comparative genomic research.

## ANNOUNCEMENT

Ants symbiotically associate with many different Actinomycetota, *Pseudonocardia,* and *Streptomyces*, which colonize their exoskeletons and participate in defense through the production of antimicrobial metabolites for the host (1–3). To investigate such microbial diversity, Actinomycetota isolated from leaf cutter ants that were collected from Dehradun, Uttarakhand, India, was identified and characterized. The ant specimens were collected on 20 December 2019, using sterile nets and transported to the lab in sterile plastic containers. Individual ants were aseptically crushed in 500 µL sterile Milli-Q water, and the resulting suspension was spread on International *Streptomyc*es Project medium 2 (ISP-2) agar (4, 5). Plates were incubated at 37°C for 5–7 days to recover Actinomycetota colonies. Isolate VRG-4 (GenBank accession no. PZ702497) showed 99.8% 16S rRNA similarity to *Streptomyces tendae* (D63873). Genome-based analyses (TYGS) confirmed its identity, with 71.6% dDDH (6), 96.63% OrthoANIu (7), and 96.88% NCBI ANI (8). Culture growth and DNA preparation were performed following the previously described method of Choudhary et al. (9). Briefly, the isolate was cultured in nutrient broth (pH 7.2) at 30°C and 180 rpm for 48 h. Genomic DNA was extracted using lysozyme, proteinase K, SDS treatment, and phenol-chloroform extraction, followed by ethanol precipitation. DNA was dissolved in nuclease-free water and stored at 4°C. Genomic DNA was quantified using Qubit 3 Fluorometer with the Qubit dsDNA High-Sensitivity Assay Kit (Thermo Fisher Scientific, India). High-quality genomic DNA (100 ng) was enzymatically fragmented to produce inserts between 200 and 300 bp. The fragmented DNA underwent end repair to produce blunt-ended fragments, followed by 3′ adenylation and ligation of Illumina-compatible loop adapters. Adapter cleavage was performed by the uracil-specific excision reagent (USER) enzyme. Adapter-ligated DNA was purified using AMPure XP beads and amplified following six cycles of PCR using NEBNext Ultra II Q5 Master Mix, Illumina universal primers, and sample-specific index primers. Amplified libraries were purified again with AMPure XP beads and eluted in 15 µL 0.1 × TE buffer. Library concentration was obtained from the Qubit 3 Fluorometer, and fragment size distribution was determined with an Agilent 2100 Bioanalyzer with a DNA 7500 chip before sequencing. Whole-genome sequencing was performed on the Illumina NovaSeq 6000 platform, creating approximately 6.2 million paired-end reads with an average read length of 151 bp. The genome was assembled *de novo* using Unicycler V.0.5.1 after quality control with FastQC V.0.12.1/MultiQC V.1.30 and adapter trimming using Trim Galore V.0.6.4. Scaffolding with Medusa V.1.6 yielded a draft genome consisting of 83 contigs (82 chromosomal and 1 plasmid), totaling 9.27 Mb, with an N50 of 453.3 kb, 92.34× average coverage, and a GC content of 72%. Genome annotation was performed using NCBI Prokaryotic Genome Annotation Pipeline (PGAP V.6.10). The annotated genome had 8,243 predicted protein-coding sequences, 69 tRNA genes, 6 rRNA genes (5S, 16S, and 23S), 1 tmRNA gene. antiSMASH v8.0.4 results showed 37 putative biosynthetic gene clusters, including those encoding NRPSs, type I–III PKSs, hybrid NRPS–PKS systems, terpenes, siderophores, lanthipeptides, RiPPs, melanin, indole, and ectoine.

## Data Availability

The draft genome sequence of *Streptomyces* sp. strain VRG-4 has been deposited in the NCBI database under BioProject accession number PRJNA1438909, BioSample accession number SAMN56551269, assembly accession number GCA_056315905.1, and SRA accession number SRX32916998.

## References

[1] Marsh SE, Poulsen M, Pinto-Tomás A, Currie CR. 2014. Interaction between workers during a short time window is required for bacterial symbiont transmission in *Acromyrmex* leaf-cutting ants. PLoS One 9:e103269. doi:10.1371/journal.pone.0103269 25058579 PMC4110003

[2] Batey SFD, Greco C, Hutchings MI, Wilkinson B. 2020. Chemical warfare between fungus-growing ants and their pathogens. Curr Opin Chem Biol 59:172–181. doi:10.1016/j.cbpa.2020.08.001 32949983 PMC7763482

[3] Van Arnam EB, Ruzzini AC, Sit CS, Horn H, Pinto-Tomás AA, Currie CR, Clardy J. 2016. Selvamicin, an atypical antifungal polyene from two alternative genomic contexts. Proc Natl Acad Sci USA 113:12940–12945. doi:10.1073/pnas.1613285113 27803316 PMC5135293

[4] Shirling EB, Gottlieb D. 1966. Methods for characterization of *Streptomyces* species. Int J Syst Bacteriol 16:313–340. doi:10.1099/00207713-16-3-313

[5] Zhang MM, Poulsen M, Currie CR. 2007. Symbiont recognition of mutualistic bacteria by *Acromyrmex* leaf-cutting ants. ISME J 1:313–320. doi:10.1038/ismej.2007.41 18043642

[6] Meier-Kolthoff JP, Göker M. 2019. TYGS is an automated high-throughput platform for state-of-the-art genome-based taxonomy. Nat Commun 10:2182. doi:10.1038/s41467-019-10210-3 31097708 PMC6522516

[7] Yoon SH, Ha SM, Lim JM, Kwon SJ, Chun J. 2017. A large-scale evaluation of algorithms to calculate average nucleotide identity. Antonie Van Leeuwenhoek 110:1281–1286. doi:10.1007/s10482-017-0844-4 28204908

[8] Ciufo S, Kannan S, Sharma S, Badretdin A, Clark K, Turner S, Brover S, Schoch CL, Kimchi A, DiCuccio M. 2018. Using average nucleotide identity to improve taxonomic assignments in prokaryotic genomes at the NCBI. Int J Syst Evol Microbiol 68:2386–2392. doi:10.1099/ijsem.0.002809 29792589 PMC6978984

[9] Choudhary M, Naik B, Kumar V, Verma A, Gupta S. 2023. Draft genome sequence of novel *Streptomyces* sp. strain AJ-1, isolated from a leafcutter ant in Uttarakhand, India. Microbiol Resour Announc 12:e00413-23. doi:10.1128/mra.00413-23 37338399 PMC10353396

